# Notes on the Mining Districts
†Report of the Commissioner appointed under the Provisions of the Act 5 and 6 Vict. c. 99, to inquire into the Operation of that Act, and into the State of the Population in the Mining Districts. Presented to both Houses of Parliament by command of Her Majesty. London: 1855.


**Published:** 1856-04

**Authors:** 


### THE EPITOME OF SANITARY LITERATURE.

### NOTES ON THE MINING DISTRICTS.†

FROM a Report (1855) by Mr. H. S. TREMENHEERE, the

---

† Report of the Commissioner appointed under the Provisions of the Act commissioner appointed under the provisions of the Act 5 and 6 Victoria, c. 99, to inquire into the operation of that Act, and into the state of the population in the mining districts, we learn that females continue to be employed in the collieries of South Wales; and, as an evidence of the degradation of these female miners, we are told that in some cases they work in men's clothes, to avoid discovery.

The employment of boys under ten years of age under ground, has also become much more general than hitherto; South Wales, Staffordshire, and Yorkshire, being the localities where they are employed to the greatest extent. So general, indeed, is this practice, that one manager is stated to have alleged, as his reason for refusing to abandon it, that "the other managers were allowing it." This occurred in a district where, on two occasions, considerable expense had been incurred in prosecutions.

The frequent and generally unreasonable strikes among colliers, their almost universal habit of working less the higher their wages are, their sacrifice of their children's interests to procure themselves more means of self-indulgence, and their gross ignorance, are facts as patent now as when the Act was passed, in 1843, and prove the necessity of educating at least the youthful portion of this large section of the population.

The author of this Report, on former occasions made suggestions on the subject of education, which were examined by a Committee of the House of Commons, who made certain objections to them. As far, however, as we can judge from the reprints before us, the education of children in the mining districts is making some amount of hopeful progress. The system of giving money prizes, originating through Mr. Tremenheere, works well. The object of the associations established for this purpose (now seven in number, five being in operation) is to offer inducements for children to attend school in the mining districts, especially children of the mining population; to remain longer at school, until they have received the rudiments of a sound education, instead of leaving school, as has been the general habit, at so early an age as to make the little they learn almost entirely useless to them hereafter. In Staffordshire alone, the candidates examined for the prizes of the year 1854-5 amounted to 619;

---

5 and 6 Vict. c. 99, to inquire into the Operation of that Act, and into the State of the Population in the Mining Districts. Presented to both Houses of Parliament by command of Her Majesty. London: 1855.

of whom 365 were boys, and 214 girls. It appears, however, from the report of the Rev. J. P. Norris, Her Majesty's Inspector of Schools for Staffordshire, that the children of colliers and miners amount to only 6 per cent. of those who presented themselves for examination under these prize schemes. Mr. Norris' report contains his conviction that "nothing short of legislative interference can redress the educational balance of these mining districts. At present the divergence between the employers and the employed is increasing every year. Every year's delay brings us nearer to a crisis."

Quotations from Mr. Herbert Mackworth, an experienced Inspector of Mines, show that the care on the part of the employer for the social and sanitary welfare of the miner is somewhat increasing; but that far greater attention is paid to these important matters on the continent. The works stretching from Valenciennes to Aix-la-Chapelle are cited in illustration; where the machinery, top of the pit, is enclosed by enormous buildings, in which are rooms for dressing, undressing, washing, clothes drying, etc. Even in 1826, the Anzin Company erected bathing halls for the miners, supplied with waste hot water from the pumping engines. Several of the continental mines have barracks, fitted for those men who only return home once a week; and an accident room, with remedies for sudden illness and accidents, and printed directions for preliminary measures are frequently to be met with.

Mr. Tremenheere states that a mining village, with gardens, schools, etc., has this year been nearly completed in Scotland, by the dowager Lady Ruthven, on her estate of Winton, East Lothian, which will nearly equal those on the continent. He found in this village lately, many remnants of prejudice and ignorance, on subjects of the first importance to their health and well-being.

We learn, finally, from this Report, that the rapidity with which miners die out, and the increasing demand for minerals, already causes difficulty in keeping up a supply of miners adequate to the demand; and the author of the Report points to this as suggesting to employers the expediency of doing all in their power to make miners satisfied with their lot, and as affording a warning against needless legislative pressure as regards the labour of their children, which might tend to divert them to other occupations.

